# Structural changes in the extracellular loop 2 of the murine KCC2 potassium chloride cotransporter modulate ion transport

**DOI:** 10.1016/j.jbc.2021.100793

**Published:** 2021-05-18

**Authors:** Anna-Maria Hartmann, Lifei Fu, Christine Ziegler, Michael Winklhofer, Hans Gerd Nothwang

**Affiliations:** 1 Division of Neurogenetics, School of Medicine and Health Sciences, Carl von Ossietzky University Oldenburg, Oldenburg, Germany; 2 Research Center for Neurosensory Sciences, Carl von Ossietzky University Oldenburg, Oldenburg, Germany; 3 Biophysics II, Biophysics II-Structural Biology, Faculty of Biology and Pre-Clinical Medicine, University of Regensburg, Regensburg, Germany; 4 Institute for Biology and Environmental Sciences IBU, Carl von Ossietzky University of Oldenburg, Oldenburg, Germany; 5 Center of Excellence Hearing4all, Carl von Ossietzky University Oldenburg, Oldenburg, Germany

**Keywords:** structure, site-directed mutagenesis, protein conformation, cation–chloride cotransporter, cell culture, CCC, cation–chloride cotransporter, ECD, extracellular domain, EL2, extracellular loop 2, KCCs, K^+^–Cl^−^ cotransporters, NKCC, Na^+^–K^+^–Cl^−^ cotransporters, TMs, transmembrane helices

## Abstract

K^+^–Cl^−^ cotransporters (KCCs) play important roles in physiological processes such as inhibitory neurotransmission and cell-volume regulation. KCCs exhibit significant variations in K^+^ affinities, yet recent atomic structures demonstrated that K^+^- and Cl^−^-binding sites are highly conserved, raising the question of whether additional structural elements may contribute to ion coordination. The termini and the large extracellular domain (ECD) of KCCs exhibit only low sequence identity and were already discussed as modulators of transport activity. Here, we used the extracellular loop 2 (EL2) that links transmembrane helices (TMs) 3 and 4, as a mechanism to modulate ECD folding. We compared consequences of point mutations in the K^+^-binding site on the function of WT KCC2 and in a KCC2 variant, in which EL2 was structurally altered by insertion of a IFYPYDVPDYAGYPYDVPDYAGSYPYDVPDYAAHAAA (3xHA) tag (36 amino acids). In WT KCC2, individual mutations of five residues in the K^+^-binding site resulted in a 2- to 3-fold decreased transport rate. However, the same mutations in the KCC2 variant with EL2 structurally altered by insertion of a 3xHA tag had no effect on transport activity. Homology models of mouse KCC2 with the 3xHA tag inserted into EL2 using *ab initio* prediction were generated. The models suggest subtle conformational changes occur in the ECD upon EL2 modification. These data suggest that a conformational change in the ECD, for example, by interaction with EL2, might be an elegant way to modulate the K^+^ affinity of the different isoforms in the KCC subfamily.

The cation–chloride cotransporter (CCC) family consists of secondary active membrane transporters mediating the symport of cations (Na^+^ and K^+^) coupled with chloride (Cl^−^). They belong to the amino acid/polyamine/organocation superfamily and therefore adopt the so-called LeuT-fold (1, 2, 3). Gene-duplication events caused diversification into four CCC subfamilies: the K^+^–Cl^−^ cotransporters (KCC1-4), the Na^+^–K^+^–Cl^−^ cotransporters (NKCC1 + 2), and Na^+^–Cl^−^ cotransporters, the CCC-interacting protein CIP1, and the polyamine transporter CCC9 (4, 5, 6). CCCs are involved in various physiological processes such as *trans*-epithelial ion absorption, cell-volume regulation, and chloride homeostasis. In line with their importance, mutations therein result in numerous human pathologies (3, 4, 5, 6, 7, 8, 9, 10, 11).

Many of the nervous system–associated pathologies are associated with dysfunction of KCC2. This transporter is the major Cl^−^ extrusion cotransporter in most neurons of the central nervous system (9, 12). Hence, the transporter has a pivotal role in the fast hyperpolarizing action of GABA and glycine as these receptors represent ligand-gated Cl^−^ channels (9, 13). Mice with disruption of the *SLC12A5* gene die shortly after birth because of motor deficits (14, 15). *SLC12*A5 encodes two splice variants, KCC2a and KCC2b, that differ in the first exon coding the distal part of the N terminus. KCC2b-deficient mice survive up to 3 weeks postnatally (16), whereas KCC2a-deficient mice show no obvious neurological phenotype (17). Dysregulation of KCC2 is associated with several neurological and psychiatric disorders including epilepsy, neuropathic pain, spasticity, ischemic insults, brain trauma, schizophrenia, and autism (18, 19, 20, 21, 22, 23, 24, 25, 26, 27, 28).

CCCs consist of 12 transmembrane helices (TMs), flanked by intracellularly located N- and C-termini and a large extracellular domain (ECD) (4, 29, 30). The termini are important for trafficking, isotonic activity, and regulation *via* post-translational modifications (6, 31, 32, 33, 34, 35, 36). The N-terminal domains of KCCs have an autoinhibitory function locking the transporter in the inward-facing state to prevent intracellular solvent access to the ion-binding sites formed by residues in TM1, 3, 6, and 8 (35, 36, 37). As in all LeuT-fold transporters, TMs 1 to 5 and TMs 6 to 10 are symmetry related and inversely orientated (5 + 5 TM inverted repeat structure) (38, 39, 40, 41, 42).

Recent three-dimensional reconstruction from single-particle cryo-EM provided insight into the highly conserved ion-binding sites in KCCs and NKCC1 (35, 36, 37, 43, 44, 45, 46, 47), suggesting a similar mechanism of Cl^−^ coupling and K^+^ transport (43, 48, 49). Although the K^+^ and Cl^−^ affinities vary among CCCs (4, 48), the coordination residues of these ion-binding sites are highly conserved. The K^+^-binding site comprises five strictly conserved amino acid residues located in TM1 (Asn133 and Ile134), TM3 (Tyr218), and TM6 (Pro432 and Thr435) (Fig. 1, annotation according to murine KCC2a used in this study). There are also two conserved Cl^−^-coordinating sites described in KCCs and NKCCs (35, 43, 48, 49) (Fig. 1*B*).

**Figure 1:**
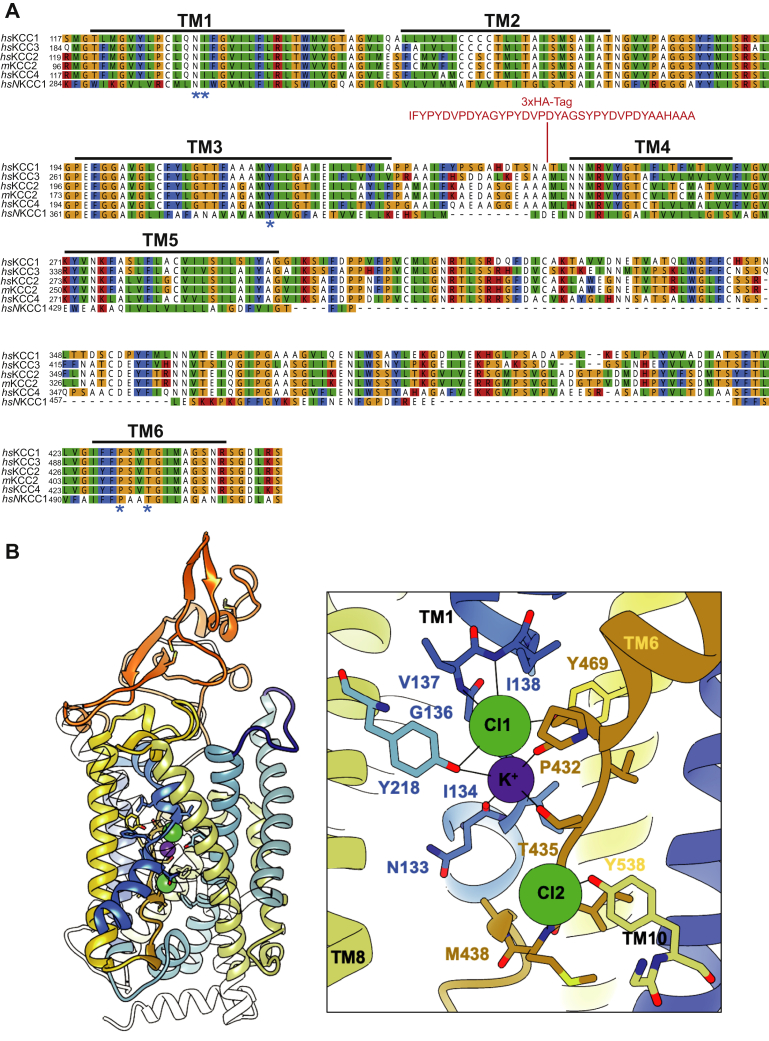
**Evolutionary conservation of K**^**+**^**-binding site in CCCs.***A*, multialignment of TM1 to TM6 of mouse KCC2 (NM_020333.2), human KCC2 (NP_001128243.1), human KCC1 (NP_005063.1), KCC3 (NP_598408.1), KCC4 (NP_006589.2), and NKCC1 (NP_001037.1) was generated using ClustalW in Geneious. The TMs and K^+^-binding sites were annotated according to the cryo-EM structure of *hs*KCC1 (47). K^+^-binding sites located in TM1 (N and I), TM3 (Y), and TM6 (P and T) are marked with an *asterisk*. In addition, analyzed residues in TM3 (Y, T, M) and TM5 (K) are marked with a *plus*. The insertion of the HA tag of KCC2b^HA^ at the end of EL2 between TM3 and TM4 is highlighted in *red*. *B*, homology model of *mm*KCC2a. All KCC structures were used for a multitemplate approach as the *hs*KCC2 structure was limited in resolution. EL2 is colored in *blue*, ECD in *orange*, TM6, 7, and 8 are colored in shades of *yellow*, and TM1, 2, 3, and 4 in *blue*. All other helices were set as transparent for better visualization. *Inset*, the conserved K^+^ site and the two Cl^−^-binding sites were modeled based on the KCC1-4 templates. The model with the best stereochemistry was chosen. The three ions are tightly coordinated mainly by the main chain of the flexible regions in the middle of TM1 and TM6 (annotation as for *mm*KCC2a, see the text). CCC, cation–chloride cotransporter; ECD, extracellular domain; EL2, extracellular loop 2; *hs*, *Homo sapiens*; KCCs, K^+^–Cl^−^ cotransporters; *mm*, *Mus musculus*; NKCC, Na^+^–K^+^–Cl^−^ cotransporters; TMs, transmembrane helices.

Cryo-EM also sheds light into the functional role of the ECD of KCCs, which extends up to ∼35 Å above the membrane. The ECD consists of a short three-stranded antiparallel beta sheet and short helices and is stabilized by two disulfide bonds (35, 36). The ECD forms an extracellular seal to the external substrate pathway (43, 46, 47). Point mutations of highly conserved cysteines in the ECD or ECD chimeras of KCC2 and KCC4 resulted in transport-inactive variants (50), suggesting that conformational changes in the ECD impact ion coordination indirectly. Here, we made use of a KCC2 variant with an 3xHA tag in the extracellular loop 2 (EL2) connecting TM3 and TM4 (KCC2^HA^). EL2 is located close to the ECD and we hypothesized that structural modification of this loop will affect folding of the ECD. While KCC2^WT^ and KCC2^HA^ showed same functionality, we observed striking differences when residues coordinating the K^+^ binding site were mutated. The structural modification of EL2 in KCC2^HA^ yielded a variant which despite mutation of conserved sites could still transport K^+^. Homology modeling suggested that insertion of the HA tag is indeed inducing conformational changes in the ECD because of a modified EL2 interaction. These changes most likely are transduced *via* the extracellular interaction network (46) of the ECD to TM1 and TM6 and seem to alter to the K^+^–binding sites in KCC2^HA^.

## Results

### KCC2a, KCC2b, and KCC2b^HA^

**Figure 2:**
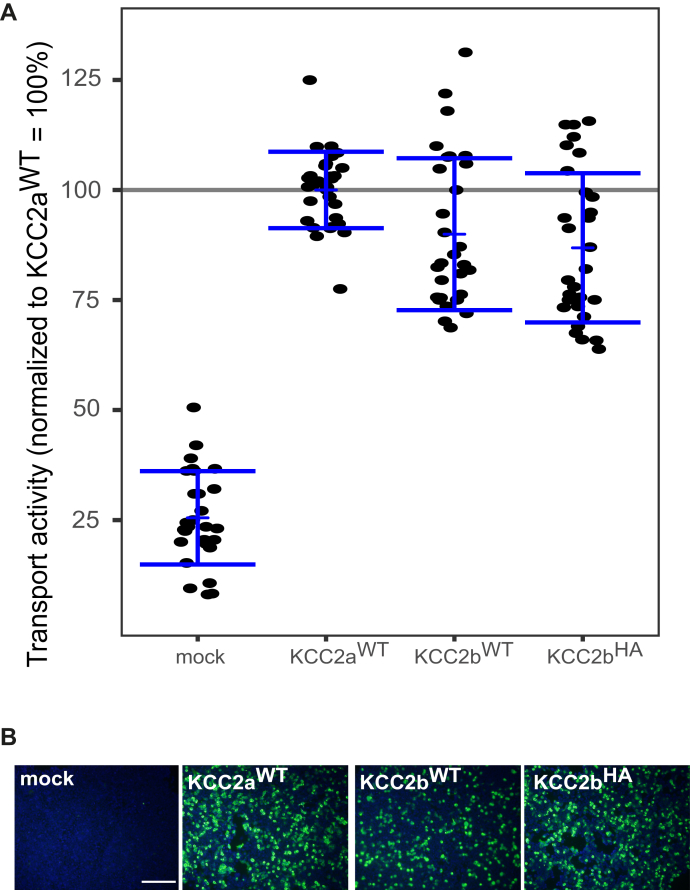
**KCC2a, KCC2b, and KCC2b**^**HA**^**are indistinguishable in their transport activity.** HEK-293 cells were transfected with KCC2a^wt^, KCC2b^wt^, or KCC2b^HA^. *A*, transport activity was determined by performing Tl^+^ flux measurements. Tl^+^ flux is indistinguishable for all three constructs: KCC2a^wt^: 100% ± 9%, KCC2b^wt^ (90% ± 17%, *p* = 0.25 compared with KCC2a^wt^), and KCC2b^HA^ (87% ± 17%, *p* = 0.1 compared with KCC2a^WT^). The graph represents the data of at least ten independent measurements including three technical replicates per independent measurement, normalized to KCC2a^wt^. Statistical analysis is presented in Table 1 (∗∗∗*p* < 0.001, Tukey's honest significant differences). The *blue bracket* represents the mean (*short bar*) ± SD. *B*, transfection rates were monitored by immunocytochemistry against the transporter (*green*) and cell staining by DAPI (*blue*). The scale bar represents 200 μm. KCCs, K^+^–Cl^−^ cotransporters.

KCC2 is present in the two splice variants KCC2a and KCC2b that differ in the first exon coding the distal part of the N terminus. To analyses whether these two splice variants differ in their transport activity, we performed Tl^+^-based flux measurements with both of them (KCC2a^WT^ and KCC2b^WT^). To investigate the impact of the ECD on KCC2-mediated transport, we used KCC2b with a 3xHA tag (IFYPYDVPDYAGYPYDVPDYAG-SYPYDVPDYAAHAAA) in EL2 of the KCC2b clone (KCC2b^HA^). The tag with a length of 37 amino acid residues is inserted at the end of EL2 reaching into TM4 (31) (Fig. 1*A*). In parallel, we monitored their transfection rates to provide similar transfection rates for every construct used in our flux measurements. Tl^+^ flux measurements demonstrated that KCC2a^WT^ (100% ± 9%), KCC2b^WT^ (90% ± 17%, *p* = 0.25), and KCC2b^HA^ (87% ± 17%, *p* = 0.1) possess similar transport activities (Fig. 2*A*, Table 1). All three constructs result in an at least 3.3 times higher transport activity than mock transfected cells (26% ± 11%) (Fig. 2*A*).

**Table 1:** Comparison of the transport activities of KCC2a^WT^, KCC2b^WT^, and KCC2b^HA^

Construct	Mean ± SD	Significance in comparison with mock	Significance in comparison with KCC2a^wt^	Significance in comparison with KCC2b^wt^	Significance in comparison with KCC2b^HA^
Mock	26% ± 11%	-	∗∗∗	∗∗∗	∗∗∗
KCC2a^wt^	100% ± 9%	∗∗∗	-	n.s.	n.s.
KCC2b^WT^	90% ± 17%	∗∗∗	n.s.	-	n.s.
KCC2b^HA^	87% ± 17%	∗∗∗	n.s.	n.s.	-

∗∗∗*p* < 0.001; n.s., not significant.

### K^+^-binding sites in KCC2a

**Figure 3:**
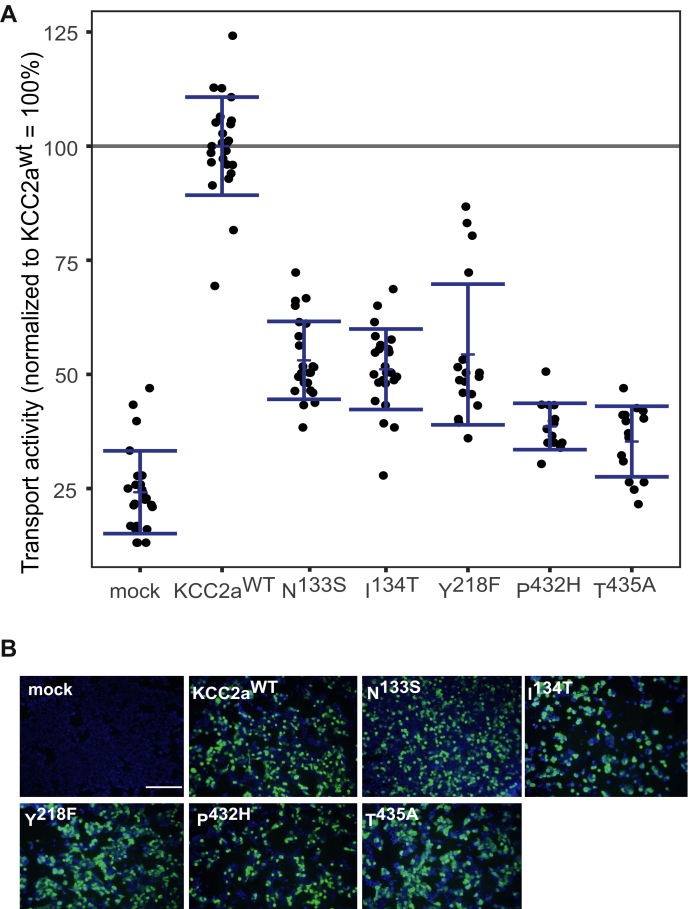
**Mutation of K**^**+**^**-binding sites diminished KCC2a activity.** HEK-293 cells were transfected with KCC2a^wt^ or KCC2 variants with mutations in the K^+^-binding site. *A*, transport activity was determined by performing Tl^+^ flux measurements. The Tl^+^ flux determined for KCC2^N133S^ (53% ± 9%, *p* = 1.44 × 10^−12^), KCC2^I134T^ (51% ± 9%, *p* = 2.33 × 10^−10^) in TM1, KCC2^Y218F^ (54% ± 15%, *p* = 9.3 × 10^−8^) in TM3, and KCC2^P432H^ (39% ± 5%, 5.3 × 10^−10^), or KCC2^T435A^ (35% ± 8%, *p* = 5.66 × 10^−10^) in TM6 showed decreased transport activity compared with that of KCC2a^wt^ (100% ± 11%). The graph represents the data of at least five independent measurements including three technical replicates per independent measurement, normalized to KCC2a^wt^. Statistical analysis is presented in Table 2 (∗∗∗*p* < 0.001, two-sample *t* test with Benjamini–Hochberg adjustment for multiple comparisons). *Blue brackets* represent the mean (*short bar*) ± SD. *B*, transfection rates were monitored by immunocytochemistry against the transporter (*green*) and cell staining by DAPI (*blue*). The scale bar represents 200 μm. KCCs, K^+^–Cl^−^ cotransporters; TM, transmembrane helix.

Recent cryo-EM structures revealed the K^+^-binding site in CCCs being formed by an asparagine and isoleucine in TM1, a tyrosine in TM3, and a proline and a threonine in TM6 (43, 46, 48, 49), in agreement with mutagenesis data for KCC1, KCC3, KCC4, and NKCC1 (36, 43, 44, 45, 46) (Fig. 1, *A* and *B*). However, resolution in the *mm*KCC2a cryo-EM structure (35) was not high enough to assign K^+^ ions. To confirm predicted K^+^ coordination in KCC2, we mutated the corresponding sites into residues of a similar size but different charge. This resulted in the following murine KCC2a mutations: KCC2a^N133S^, KCC2a^I134T^ (TM1), KCC2a^Y218F^ (TM3), and KCC2a^P432H^, and KCC2a^T435A^ (TM6). All mutants showed transfection rates in HEK-293 cells similar to KCC2a^wt^ (Fig. 3*B*). Tl^+^ flux measurements revealed significantly reduced transport activity for each of the five mutants by an average of 53% ± 9% for KCC2a^N133S^ (*p* = 1.44 × 10^−12^), 51% ± 9% for KCC2a^I134T^ (*p* = 2.33 × 10^−10^), 54% ± 15% for KCC2a^Y218F^ (*p* = 9.3 × 10^−8^), 39% ± 5% for KCC2^P432H^ (*p* = 5.3 × 10^−10^), and 35% ± 8% for KCC2^T435A^ (*p* = 5.66 × 10^−10^) compared with KCC2a^wt^ (100% ± 9%) (Fig. 3*A*; Table 2). These data confirm the K^+^ coordination and its impact on KCC2a transport activity and corroborate the conservation of the K^+^-binding site across CCCs.

**Table 2:** Transport activity of K^+^-binding site mutations in KCC2a

Construct	Mean ± SD	Significance in comparison with KCC2a^wt^	Significance in comparison with mock
Mock	24% ± 9%	∗∗∗	-
KCC2a^wt^	100% ± 11%	-	∗∗∗
N^133S^	53% ± 9%	∗∗∗	∗∗∗
I^134T^	51% ± 9%	∗∗∗	∗∗∗
Y^218F^	54% ± 15%	∗∗∗	∗∗∗
P^432H^	39% ± 5%	∗∗∗	∗∗∗
T^435A^	35% ± 8%	∗∗∗	∗∗

∗∗*p* < 0.01; ∗∗∗*p* < 0.001.

### K^+^-binding sites in KCC2b

**Figure 4:**
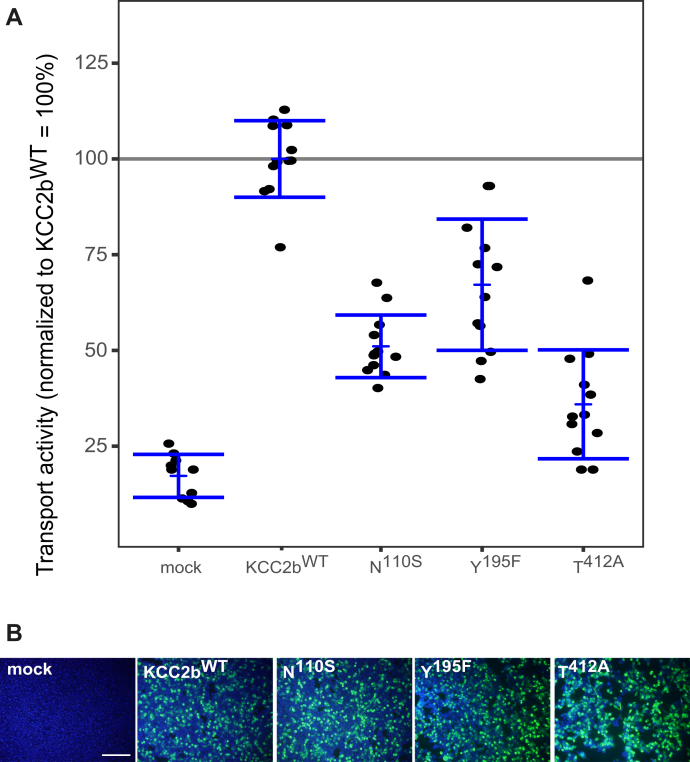
**Mutation of K**^**+**^**-binding sites diminished KCC2b activity.** HEK-293 cells were transfected with KCC2b^wt^ or KCC2 variants with mutations in the K^+^-binding site. *A*, transport activity was determined by performing Tl^+^ flux measurements. The Tl^+^ flux determined for KCC2^N110S^ (51% ± 8%, *p* = 1.89 × 10^−5^) in TM1, KCC2^Y195F^ (67% ± 17%, *p* = 0.0012) in TM3, or KCC2^T412A^ (36% ± 14%, *p* = 1.89 × 10^−5^) in TM6 showed decreased transport activity compared with that of KCC2b^wt^. The graph represents the data of at least four independent measurements including three technical replicates per independent measurement, normalized to KCC2b^wt^. Statistical analysis is presented in Table 3 (∗∗∗*p* < 0.001, two-sample *t* test with Benjamini–Hochberg adjustment for multiple comparisons). *Blue brackets* represent the mean (*short bar*) ± SD. *B*, transfection rates were monitored by immunocytochemistry against the transporter (*green*) and cell staining by DAPI (*blue*). The scale bar represents 200 μm. KCCs, K^+^–Cl^−^ cotransporters; TM, transmembrane helix.

To analyze whether these K^+^-binding sites are also important in KCC2b^WT^, we generate the following analogous KCC2b mutations (annotation shifted because of a 23 aa shorter N-terminal domain compared with KCC2a): KCC2b^N110S^ in TM1, KCC2b^Y1958F^ in TM3, and KCC2b^T412A^ in TM6. All mutants showed a transfection rate in HEK-293 cells similar to KCC2b^wt^ (Fig. 4*B*). Tl^+^ flux measurements revealed similar reduced transport activity for the each of the three mutants by an average of 51% ± 8% for KCC2b^N110S^ (*p* = 1.89 × 10^−5^), 67% ± 17% for KCC2b^Y195F^ (*p* = 0.0012), and KCC2b^T412A^ (36% ± 14%, *p* = 1.89 × 10^−5^) compared with KCC2b^wt^ (100% ± 10%) (Fig. 4*A*; Table 3). These data demonstrate that the predicted K^+^ coordination sites in KCC2a and KCC2b are important for KCC2 transport activity.

**Table 3:** Transport activity of K^+^-binding site mutations in KCC2b

Construct	Mean ± SD	Significance in comparison with KCC2b^wt^	Significance in comparison with mock
Mock	17% ± 6%	∗∗∗	-
KCC2^wt^	100% ± 10%	-	∗∗∗
N^110S^	51% ± 8%	∗∗∗	∗∗∗
Y^195F^	67% ± 17%	∗∗	∗∗
T^412A^	36% ± 14%	∗∗∗	∗

∗*p* < 0.05; ∗∗*p* < 0.01; ∗∗∗*p* < 0.001.

### Extracellular HA tag in EL2 impacts KCC2 K^+^-binding site

**Figure 5:**
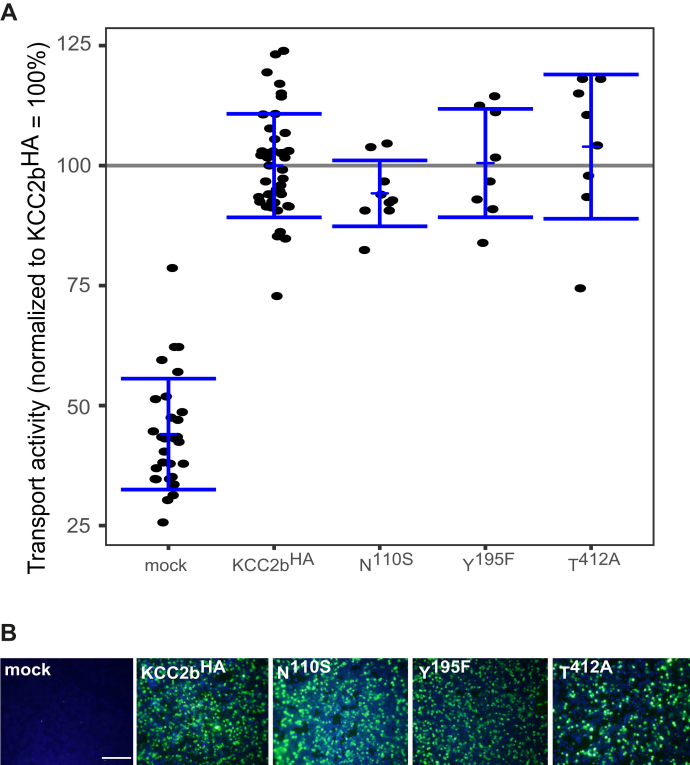
**Mutation of the K**^**+**^**-binding site do not alter KCC2**^**HA**^**activity.** HEK-293 cells were transfected with KCC2b^HA^ or KCC2 variants in which the K^+^-binding sites were mutated. *A*, transport activity was determined by performing Tl^+^ flux measurements. The Tl^+^ flux determined for KCC2^HA-N110S^ (94% ± 7%, *p* = 0.29) in TM1, KCC2^HA-Y195F^ (101% ± 11%, *p* = 0.90) in TM3, or KCC2^HA-T412A^ (104% ± 15%, *p* = 0.51) in TM6 was similar to the flux of KCC2^wt^ (100% ± 11%). The graph represents the data of at least three independent measurements including three technical replicates per independent measurement, normalized to KCC2^HA^. Statistical analysis is presented in Table 4 (∗∗∗*p* < 0.001, two-sample *t* test with Benjamini–Hochberg adjustment for multiple comparisons). *Blue brackets* represent the mean (*short bar*) ± SD. *B*, transfection rates were monitored by immunocytochemistry against the transporter (*green*) and cell staining by DAPI (*blue*). The scale bar represents 200 μm. KCCs, K^+^–Cl^−^ cotransporters; n.s., not significant; TM, transmembrane helix.

Having confirmed the conservation of the K^+^-binding site in KCC2, we next made use of a previously reported KCC2b clone with an extracellular 3xHA tag (KCC2^HA^) between TM3 and TM4. The tag with a length of 37 amino acid residues is inserted at the very end of EL2 in front of TM4 (31) (Fig. 1*A*). Importantly, this variant supports transport activity indistinguishable from KCC2b^WT^ (Fig. 2*A*). We first probed whether the insertion has a direct impact on the coordinating tyrosine residue in TM3, which is directly connected to EL2. Replacement of this tyrosine (Y195 in KCC2b^HA^) by phenylalanine had no effect on KCC2b^HA^ transport activity (Fig. 5*A*, Table 4). We next investigated whether coordinating amino acid residues in the other two TMs, involved in K^+^ binding, were also affected. We therefore mutated asparagine in TM1 (KCC2b^HA-N110S^) and threonine (KCC2b^HA-T412A^) in TM6. Again, these mutants showed flux activities indistinguishable from KCC2b^HA^ (Fig. 5*A*, Table 4). Thus, modification of EL2 by insertion of the HA-tag directly impacts K^+^ transport mechanism in KCC2b.

**Table 4:** Transport activity of K^+^-binding site mutations in KCC2b^HA^

Construct	Mean ± SD	Significance in comparison with KCC2b^HA^	Significance in comparison with mock
Mock	44% ± 12%	∗∗∗	-
KCC2^HA^	100% ± 11%	-	∗∗∗
N^110S^	94% ± 7%	n.s.	∗∗∗
Y^195F^	101% ± 11%	n.s.	∗∗∗
T^412A^	104% ± 15%	n.s.	∗∗∗

∗∗∗*p* < 0.001; n.s., not significant.

### KCC2b^HA^ interacts with the ECD and locally effects KCC2 conformation

**Figure 6:**
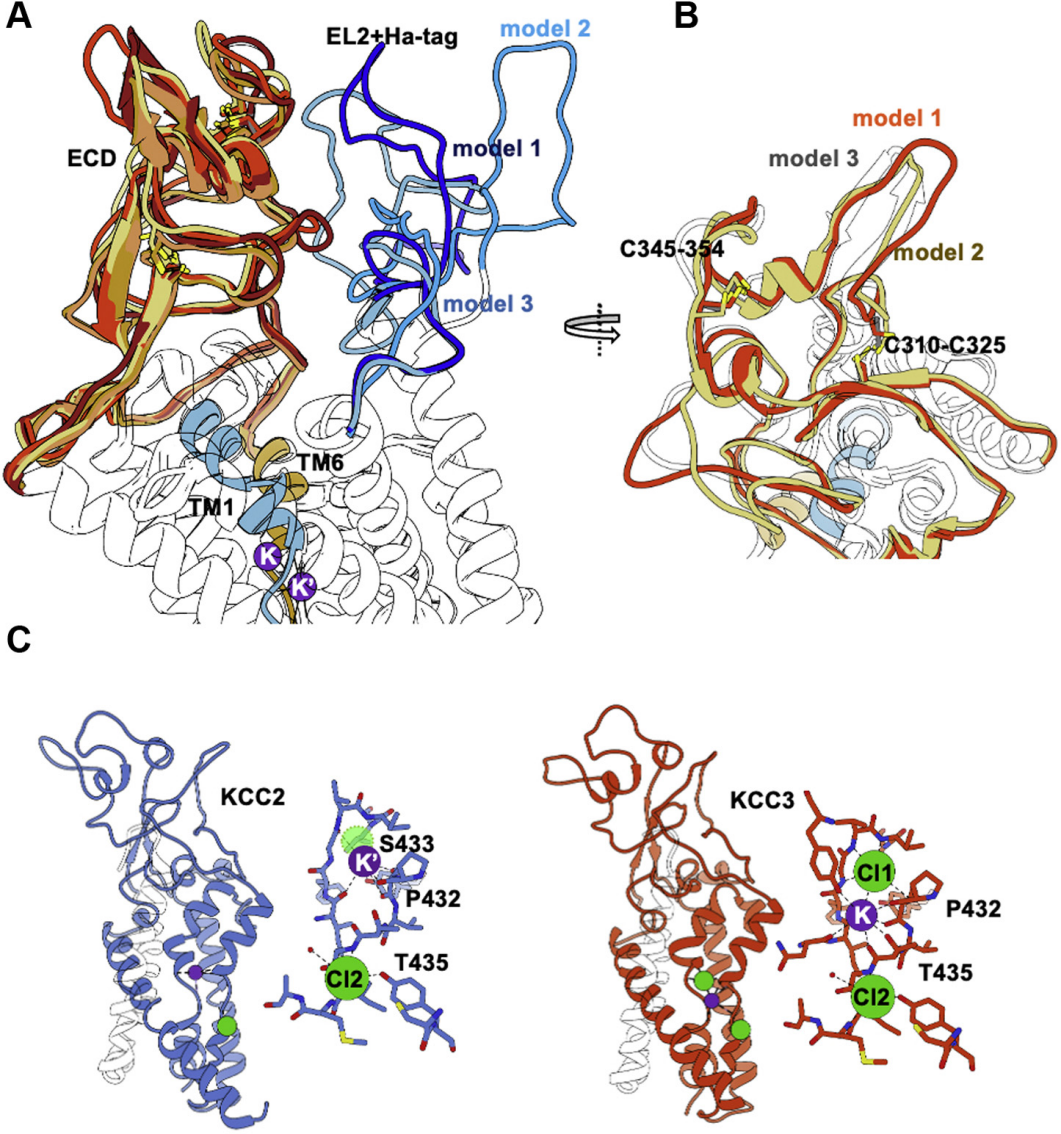
**Three-dimensional modeling of KCC2**^**HA**^**.***A*, superposition of the four best models of *mm*KCC2 with the HA tag inserted in EL2 between TM3 and TM4. The ECDs are colored in shades of *orange*-*red*, and the HA tag + EL2 is colored in *blue*. TM1 and TM6 are represented as *blue helix* and *golden helix*, respectively. Both K^+^ sites, the conserved site (K) and the alternative site (K’), are depicted. *B*, the view on the ECD for three models to visualize the conformational changes along the two disulfide bonds. *C*, ion binding in *hs*KCC2 (6m23) and *hs*KCC3 (6m1y) reveals the key role of Thr435 (annotation as for *mm*KCC2a) in coupled binding of Cl^−^ and K^+^ most likely to shape the flexible regions of TM1 and TM6 during alternating access cycling. The former CL1 site is shown as a *transparent green sphere* in KCC2 partially overlapping with the alternative K^+^ site. ECD, extracellular domain; EL2, extracellular loop 2; *hs*, *Homo sapiens*; KCCs, K^+^–Cl^−^ cotransporters; *mm*, *Mus musculus*; TMs, transmembrane helices.

To understand the impact of EL2 modification on K^+^ transport in KCC2b, we generated homology models, in which the 3xHA tag was inserted and modeled *ab initio* using the Modeller program suite. We used *hs*KCC2/hsKCC3/hsKCC4 structures (PDB codes: 6m23 (*hs*KCC2), 7d99 (*hs*KCC4), 6y5r (*hs*KCC3), 6y5v (*hs*KCC3), and 6m1y (*hs*KCC3)) to determine a set of homology models of mouse KCC2b (Fig. 1*B*). We included the 3xHA tag between Ala225 and Met226. Moreover, we included K^+^ and Cl^−^ from KCC2-4 during homology modeling according to our mutagenesis study. We created more than 30 models in an inward-facing state, which were ranked based on energy minimalization. From the best scoring models, we selected four models which showed significant variations in their ECD conformation (Fig. 6, *A* and *B*). Insertion of the HA tag did neither alter the overall conformation of *mm*KCC2 nor the coordination of K^+^ and Cl^−^ (Figs. 1*B* and 6*C*). This is in agreement with the functional data showing that the HA-tag KCC2 exhibits WT activity (Fig. 2). In the homology model of *mm*KCC2, no interaction between the ECD and EL2 is observed (Fig. 1*B*). The orientation and conformation of the HA tag changed significantly within the different homology models (Fig. 6, *A* and *B*). In all models, the EL2 + HA-tag approaches the ECD, thereby introducing subtle changes in its overall fold. Changes in the ECD fold were especially pronounced between the two cysteine bridges (Fig. 6*B*). In the model exhibiting the closest interaction with the HA tag (model 1, Fig. 6*B*), the disulfide bridge Cys310–Cys325 was even broken.

## Discussion

Recent breakthroughs in structural analysis of CCCs by cryo-EM revealed evolutionary highly conserved K^+^- and Cl^−^-binding sites in KCC1 to KCC4, and NKCC1 (35, 43, 44, 45, 46, 48, 49). Mutation of residues that coordinate the K^+^-binding site in KCC1 (Tyr216 in TM3), KCC3 (Tyr283 in TM3, Thr497 in TM6), KCC4 (Asn131 in TM1, Tyr216 in TM3), and NKCC1 (Tyr305 in TM3) diminishes or abolishes transport activity (43, 44, 46). Here, we show that mutation of the corresponding residues (Asn133 and Ile134 in TM1, Tyr218 in TM3, Pro434 and Thr435 in TM6, annotation for *mm*KCC2a) significantly reduced activity in both splice variants. Reduction of transport activity can result from intrinsic impairments or changes in post-translational modifications that affect transport activity or subcellular distribution. In the future, more in-depth analyses are necessary to determine the precise contribution of each of these putative mechanisms.

The high degree in sequence conservation raises the question why binding and transport affinities differ significantly within not only the CCC family but also within the KCC subfamily (4, 48). In brief, the affinities for K^+^ vary between 5 and 50 mM within the KCC subfamily (rabbit KCC1: >50 mM (51), rat KCC2: 5.2 ± 0.9 mM (52), human KCC3a: 9.5 ± 1.4 mM (53), mouse KCC4: 17.5 ± 2.7 mM (4) analyzed in transfected HEK-293 cells). One most likely explanation is that ion coupling, for example, the coordination of K^+^ and Cl^−^ is affected by additional structural elements contributing to the otherwise highly conserved coupling mechanism (48). This could be achieved by, for example, accessibility of the ion-binding sites or autoinhibitory effects such as recently shown for the N-terminal domain in KCCs (35, 36, 37). Another mechanistic hot spot would be the large ECD, which is intertwined with basically the entire transporter core at the extracellular side and discussed as extracellular seal. We recently demonstrated that alteration of the structural integrity of the ECD by mutagenesis dramatically affects transport in KCC2. Mutation of all four evolutionarily conserved cysteines reduced KCC2 transport activity, suggesting that the overall folding of this domain is essential for conformational changes in the transport cycle (50). The functional impact of these cysteine mutations is likely caused by the disruption of the interaction network between the TM region and ECD (Fig. 6*B*). In a similar context, mutation of the glycosylation site Asn312 in the ECD reduces *mm*KCC4 activity (43).

However, with our previous approaches of disrupting the ECD integrity, it was not possible to draw a conclusive line from the ECD fold to modified K^+^ transport. Here, we chose another approach based on our hypothesis that other extracellular loops might affect ECD folding. We decided to use a KCC2 construct in which a 37-aa-long HA tag as noninvasive alteration in EL2 was integrated. EL2 is a small loop not interacting with the ECD in KCC2^WT^ (Fig. 1*B*). The KCC2b^HA^ construct had the tremendous advantage of exhibiting WT transport activity, which allowed for a systematic investigation of the K^+^-binding sites by mutagenesis. Our modeling experiments confirmed that HA-tag insertion did not alter the overall structure or the dimerization of KCC2. The models suggest that the HA tag extends away from the dimer center, whereas in some of the models, EL2 moves into the interaction range to the ECD. We observed changes in the ECD especially in the region flanked by the two stabilizing disulfide bridges. Interestingly, this region of the ECD shows the lowest sequence conservation. Our substitution experiments of K^+^-coordinating residues in KCC2b^wt^ or KCC2b^HA^, respectively, revealed a striking effect of the HA insertion. Mutations in K^+^-binding sites, which had an unambiguous effect on transport activity in both splice variants, did not change transport activity when EL2 was prolonged by the HA tag in KCC2b^HA^. This observation suggests that the coupling mechanism that underly the K^+^ transport is affected by subtle conformational changes of the ECD.

At this point, we can only speculate why K^+^ can be transported in KCC2b^HA^ when the conserved K^+^ coordination site is corrupted. Despite the electroneutrality of the transport in KCCs, one potassium and two chloride ions are observed in most KCC structures, which were also included into our homology modeling (Fig. 1*B*). The first coordinating site (CL1) exploits conserved residues mainly in TM1 (Gly136, Val137, and Met138) (35, 43, 48, 49), and the second site (CL2) engages main chain interactions from TM6 (Gly436, Ile437, Met438) and Tyr538 in TM10 (Fig. 1*B*) (35, 36, 43, 48). However, one *hs*KCC2 structure (PDB entry code 6m23) reveals an alternative coordination for K^+^ (K’) (Fig. 6*C*, left), which is close to the Cl1 site in KCC3 (Fig. 6*C*, right; transparent green sphere in KCC2). Such an alternative site would be easily achieved considering that K^+^ and two Cl^−^ ions, respectively, are coordinated *via* a main chain network in the flexible regions of TM1 and TM6 (Fig. 1*B*). For chloride binding, main chain nitrogen of the flexible regions in TM1 and TM6 wrap around these ions.

Critical for coupled ion binding in transport to the conserved site (Figs. 1*B* and 6*C*) is Thr435, which coordinates K^+^ by its side chain and Cl^−^ by its main chain. In comparison, in the alternative site, the main chain oxygens now coordinate K^+^ together with Pro432 and Ser433 in TM6 and Tyr469 in TM7. This replaces the former coordination by Asn133, Tyr218, and Thr435, which we have mutated in our study. Obviously, Thr435 is no longer coordinating both ions simultaneously, which might affect the conformational flexibility of TM1 and TM6 at this position. To switch to an alternative K^+^ coordination, only subtle changes in TM1 and/or TM6 are required. It is possible that the interaction of HA tag in EL2 with the ECD can serve this purpose. In such a scenario, mutation of the conserved sites might have no effect when the alternative sites are present due to conformational changes in TM1 and TM6.

In a recent publication on the structure of monomeric KCC4 in nanodiscs (43), an electrostatic interaction network is described between extracellular loops including the ECD and TM1/TM6, which are involved in ion coordination. It was argued that opening of the extracellular gate in the course of alternating access requires a disruption or 'unzipping' of this electrostatic network, and the authors suggested a rotation of loop EL4 and the ECD away from the TMs. An interaction between TM6 and ECD would also be affected when a glycosylation site (Asn312 in KCC4) is mutated (43). We see here similarities to our data. The structures of KCC2 and KCC3 used for our homology models (Fig. 6*C*) show that ion-binding sites, both for K^+^ and Cl^−^, depend on several main chain coordination in TM1 and TM6. Changes in ion coordination therefore require main chain movements of TM1 and TM6. Following a similar line of argumentation as for KCC4, we hypothesize that TM1 and TM6 move because of changes in the fold of the ECD by HA-tag insertion to cause a switch from a conserved K^+^-binding site (Thr435, Pro432, Tyr218 plus main chain) to an alternative site (Pro432, Ser433, Tyr469 plus main chain). This would explain why our point mutations in the conserved site did not inactivate KCC2b^HA^. Future experiments have to clarify whether these alternative K^+^-binding sites are important in ion translocation by using site-directed mutagenesis and activity measurements.

In summary, our data suggest that conformational changes in the ECD by interactions with other extracellular loops might trigger the coordination switch, supporting the notion of a regulatory role for the ECD in K^+^–Cl^−^ cotransport.

## Experimental procedures

### Construction of expression clones

Site-directed mutagenesis of mouse KCC2a (NM_001355480.1) and mouse KCC2b (NM_020333.2) with an HA tag in EL2 and rat KCC2b (NM_134363.1) was performed according to the QuikChange mutagenesis system (Stratagene) (31, 54, 55). Forward oligonucleotides for the generation of the mutations are given in Table 5. Confirmations of the generated clones were done by sequencing (LGC genomics).

**Table 5:** Forward primers used for site-directed mutagenesis in *mm*KCC2a, *rn*KCC2b, and *mm*KCC2b

Constructs	Sequence 5’ to 3’
*mm*KCC2a/b–N133S/N110Sfor	CGTGCCTGCAGAGCATCTTTGGTGTC
*rn*KCC2–N110Sfor	CGTGCCTGCAGAGCATCTTTGGTGTT
*mm*KCC2a–I134Tfor	GCCTGCAGAACACCTTTGGTGTCATC
*mm*KCC2a/b–Y218F/Y195for	CTGGGGCTATGTTCATCCTTGGCACG
*rn*KCC2–Y195Ffor	CTGGGGCTATGTTCATCCTGGGCACC
*mm*KCC2a_P432Hfor	GGTATCTACTTCCACTCAGTCACAGGG
*mm*KCC2a/b–T435A/T412Afor	CTACTTCCCCTCAGTCGCAGGGATCATGGCTG
*rn*KCC2–T412Afor	CTATTTCCCCTCAGTCGCAGGGATCATGGCTG

for, forward; mm, *Mus musculus*; rn, *Rattus norvegicus*.

### Cell culturing

For immunocytochemistry and measurements of K^+^–Cl^−^ cotransporter activity, HEK-293 cells were transiently transfected with the respective construct, using TurboFect (Fermentas). Four hour before transfection, the medium was replaced by Dulbecco's modified Eagle's medium. For transient transfection in a 6-well plate, 6-μl TurboFect (Fermentas), 150 μl Opti-MEM (Invitrogen), and 3-μg DNA were mixed and incubated for 20 min at room temperature (RT) before transfection. For K^+^–Cl^−^ cotransporter activity measurements, HEK-293 cells were plated in a 0.1 mg/ml poly-L-lysine–coated black-well 96-well culture dish (Greiner Bio-One) at a concentration of 100,000 cells/well, 24 h after transfection. The remainders of the cells were plated on a 0.1 mg/ml poly-L-lysine–coated glass coverslip. After ∼18 h, coverslips were proceeded for immunocytochemical analyses to determine the transfection rates, which were routinely between 20 and 30%.

### Immunocytochemistry

For immunocytochemistry, all steps were performed at RT. HEK-293 cells grown on poly-L-lysine–coated coverslips were fixated for 10 min with 4% paraformaldehyde in 0.2 M phosphate buffer. After three washing steps in PBS, cells were incubated with the blocking solution (2% bovine serum albumin and 10% goat serum in PBS) for 30 min. The primary antibody solution (mouse anti-HA.11; 1:1000; Covance) was added in the carrier solution (0.3% Triton X-100, 1% bovine serum albumin, 1% goat serum in PBS) for 1 h. After washing three times in PBS, the secondary antibody, which was conjugated to a fluorescent probe (Alexa Fluor 488 goat anti-mouse; 1:1000; Thermo Fisher), was incubated for 1 h. After three washes in PBS, cells were mounted onto glass slides with Mowiol (Roth) and 4′,6-diamidin-2-phenylindol (1:1000; Roth). Photomicrographs were taken using a BioZero fluorescence microscope (Keyence).

### Determination of K^+^–Cl^−^ cotransport

Transport activity of KCC2 was determined by Cl^−^-dependent uptake of Tl^+^ in HEK-293 cells as described previously (50, 54, 56). To initiate the flux measurement, the medium in the 96-well culture dish was replaced by 80-μl hypotonic preincubation buffer (100 mM N-methyl-D-glucamine-chloride, 5 mM Hepes, 5 mM KCl, 2 mM CaCl_2_, 0.8 mM MgSO_4_, 5 mM glucose, pH 7.4; osmolarity: 175 mmol/kg ± 2 mmol/kg) with 2 μM FluoZin-2 AM dye (Invitrogen) plus 0.2% (wt/vol) Pluronic F-127 (Invitrogen) and incubated for 48 min at RT. Cells were then washed three times with 80-μl preincubation buffer and incubated for 15 min with 80 μl preincubation buffer plus 0.1 mM ouabain to block the activity of the Na^+^/K^+^ ATPases. Afterward, the 96-well plate was placed into a fluorometer (Fluoroskan Ascent, Thermo Scientific), and each well was injected with 40 μl 5x thallium stimulation buffer (12 mM Tl_2_SO_4_, 100 mM N-methyl-D-glucamin, 5 mM Hepes, 2 mM KCl, 2 mM CaCl_2_, 0.8 mM MgSO_4_, 5 mM glucose, pH 7.4). The fluorescence was measured in a kinetic-dependent manner (excitation 485 nm, emission 538 nm, one frame in 5 s in a 200-s time span) across the entire cell population in a single well. By using linear regression of the initial values of the slope of Tl^+^-stimulated fluorescence increase, the transport activity was calculated.

### Statistical analyses

Transport activities of each of the mutants were tested against the control sample (mock and WT), using two-sample *t* test after Student's *t* test for similar variances between samples or the two-sample *t* test after Welch in the few cases where SDs differed by more than a factor of two (57). Because three technical replicates were measured for each independent preparation, we deflated the number of degrees of freedom according to the actual sample size (the number of independent preparations) so as to avoid pseudoreplication. The resulting *p*-values were corrected using the Benjamini–Hochberg method (58), which controls the false-discovery rate in multiple comparisons. Note that only *p*-values <0.01 were considered to reduce the chances of false positives (type I errors). For crosswise paired comparisons of means of transport activities among KCC2a^wt^, KCC2b^wt^, KCC2b^HA^, and mock (Table 1), we computed honest significant differences according to Tukey–Kramer, using the function stats::TukeyHSD (59).

### Modeling of KCC2b^HA^

For the comparative modeling of KCC2b^HA^, five templates with the PDB entry 6y5r, 6y5v, 7d99, 6m1y, and 6m23 were selected according to the quality estimation by Global Model Quality Estimation and quaternary structure quality estimate. The template structures all adopt an inward-facing conformation. The generated models were constructed as homodimers. Homology modeling and model refinement for KCC2 with an additionally inserted 3xHA tag IF-YPYDVPDYAG-YP-YDVPDYAG-S-YPYDVPDYAAHAAA was performed in Modeller 9.12 in UCSF Chimera 1.14 individually for each template. Multitemplate alignment with position-specific scoring matrix was done in Jalview. From the outputs of 30 models, the final model with the lowest evaluation scores of normalized Discrete Optimized Protein Energy was selected for further refinement. For the no-template aligned part, the additional N-terminal and C- terminal of amino acids were deleted and nonconserved loop areas were optimized by loop modeling. The clash score was reduced by doing an energy minimization step and optimizing the rotamers of side chains. The homodimers were manually realigned in the two-fold axis between the monomers according the superposition with the template dimer. The final refinement and validation of model was done in the Phenix and Coot based on the Ramachandran plot and MolProbity score.

## Data availability

All relevant data are available from the corresponding author upon reasonable request.

## Conflict of interest

The authors declare that they have no conflicts of interest with the contents of this article.
